# Nutrient sink limitation constrains growth in two barley species with contrasting growth strategies

**DOI:** 10.1002/pld3.94

**Published:** 2018-11-12

**Authors:** Angela C. Burnett, Alistair Rogers, Mark Rees, Colin P. Osborne

**Affiliations:** ^1^ Department of Animal and Plant Sciences University of Sheffield Sheffield UK; ^2^ Environmental and Climate Sciences Department Brookhaven National Laboratory Upton New York; ^3^ Present address: Environmental and Climate Sciences Department Brookhaven National Laboratory Upton New York 11973

**Keywords:** barley, crop, growth, nitrogen, nutrients, source‐sink

## Abstract

Mineral nutrients exert important limitations on plant growth. Growth is limited by the nutrient source when it is constrained by nutrient availability and uptake, which may simultaneously limit investment in photosynthetic proteins, leading to carbon source limitation. However, growth may also be limited by nutrient utilization in sink tissue. The relative importance of these processes is contested, with crop and vegetation models typically assuming source limitations of carbon and mineral nutrients (especially nitrogen). This study compared the importance of source and sink limitation on growth in a slower‐growing wild perennial barley (*Hordeum bulbosum*) and a faster‐growing domesticated annual barley (*Hordeum vulgare*), by applying a mineral nutrient treatment and measuring nitrogen uptake, growth, allocation, and carbon partitioning. We found that nitrogen uptake, growth, tillering, shoot allocation, and nitrogen storage were restricted by low nutrient treatments. Multiple lines of evidence suggest that low nutrient levels do not limit growth via carbon acquisition: (a) Carbohydrate storage does not increase at high nutrient levels. (b) Ratio of free amino acids to sucrose increases at high nutrient levels. (c) Shoot allocation increases at high nutrient levels. These data indicate that barley productivity is limited by the capacity for nutrient use in growth. Models must explicitly account for sink processes in order to properly simulate this mineral nutrient limitation of growth.

## 1 INTRODUCTION

The use of fertiliser in agriculture is an increasing cause of concern from both economic and environmental perspectives (Masclaux‐Daubresse et al., 2010). In plants, after carbon, nitrogen is the element required in largest quantities (Sakakibara, Takei, & Hirose, 2006) and is therefore the primary mineral nutrient and a vital resource that affects growth, allocation and phenology (Stitt & Krapp, 1999). Crops are routinely treated with NPK fertiliser, containing the macronutrients nitrogen, phosphorus, and potassium. Fertiliser use has greatly improved crop yields, but the production and excess application of mineral nutrient fertiliser have simultaneously decreased agricultural nitrogen use efficiency and increased environmental damage (Erisman, Sutton, Galloway, Klimont, & Winiwarter, 2008). For instance, the manufacture of nitrogen fertiliser via the Haber‐Bosch process and its denitrification in soils are major sources of anthropogenic greenhouse gas emissions in food production (Goucher, Bruce, Cameron, Lenny Koh, & Horton, 2017), whilst agricultural run‐off has led to devastating eutrophication (fuelled by nitrogen and phosphorus) in areas such as the Gulf of Mexico (Rabalais, Turner, & Wiseman, 2002). Appropriate fertiliser application will reduce the financial, environmental, and ecological costs of intensive farming, yet the crop requirement for nutrients must be met in order to maintain and increase yields. Nutrient use efficiency is therefore a major target for crop improvement, with nitrogen being the key element of interest (Perchlik & Tegeder, 2018; Tegeder & Masclaux‐Daubresse, 2018). The timing of fertiliser application has been extensively researched from an agronomic perspective, and much is known at the molecular level about nitrate transport and signalling mechanisms (Kiba, Kudo, Kojima, & Sakakibara, 2011; Miller, Fan, Orsel, Smith, & Wells, 2007; Sakakibara et al., 2006). However, the whole‐plant physiological behaviour linking the ecology of crops on one hand, and molecular physiology on the other, remains poorly characterised.

The relationships between sources and sinks are vital determinants of growth (Chang & Zhu, 2017; White, Rogers, Rees, & Osborne, 2016). Source strength may be defined as the capacity of the plant to take up a resource from the external environment—in this case, mineral nutrients from the soil—whilst sink strength is the internal capacity of the plant to utilize that resource in storage or growth. In each case, strength is the product of the size and activity of the source or sink tissue (Geiger & Shieh, 1993; White et al., 2016). For the primary mineral nutrient nitrogen, the source:sink ratio is regulated by interacting molecular mechanisms, yet our understanding of how, and the extent to which, nitrogen source and sink strengths limit growth at different times during ontogeny remains incomplete (White et al., 2016). Broadly speaking, plants are thought to transit from carbon sink to source limitation during development (Arp, 1991; Marschner, 1995), but the generality of this principle is unclear because differences in carbon source and sink limitation have been observed for a variety of crops, particularly at grain filling (Acreche & Slafer, 2009; Álvaro, Royo, García del Moral, & Villegas, 2008; Jaikumar, Snapp, Flore, & Loescher, 2014; Peterhansel & Offermann, 2012; Slewinski, 2012). Many studies focus on reproductive growth in order to examine source‐sink relations with respect to yield, such that understanding of source‐sink relations is especially deficient for the vegetative stage (Burnett, Rogers, Rees, & Osborne, 2016). The majority of studies focus on sources and sinks for carbon which are of great importance (Paul, Oszvald, Jesus, Rajulu, & Griffiths, 2017) yet nutrient sources and sinks are critical factors that interact with carbon sources and sinks (Ruiz‐Vera, De Souza, Long, & Ort, 2017) and underpin plant growth in their own right (Sonnewald & Fernie, 2018; White et al., 2016).

Growth is controlled by a network of processes, the strengths of which vary with nutrient supply (White et al., 2016). Therefore, understanding how nutrient uptake and utilization limits growth is important for improving crop yield and sustainability (Burnett et al., 2016; White et al., 2016). In particular, the processes responsible for nutrient limitation of growth are contested. For example, low nitrogen uptake by the root could primarily constrain growth by one of two mechanisms: limiting the synthesis of photosynthetic proteins and thereby causing carbon source limitation; or directly limiting the synthesis of proteins and other compounds required for sink tissue expansion (Fatichi, Leuzinger, & Korner, 2014; Körner, 2015; Poorter, Anten, & Marcelis, 2013; Stitt & Schulze, 1994). Process‐based vegetation and crop models often represent nitrogen limitation by focusing solely on root nitrogen uptake and nitrogen use in photosynthesis (Bao, Hoogenboom, McClendon, & Vellidis, 2017; Oleson et al., 2010; Wärlind, Smith, Hickler, & Arneth, 2014), without considering the direct limiting effects of nitrogen availability on tissue growth. However, there are several exceptions (Zaehle et al., 2014).

The natural diversity of plant growth rates can be used to investigate the factors that limit growth, and ecological research has advanced our understanding of how growth and nutrient use are adapted to different soil conditions. Growth rate is considered an important adaptation to variation in soil fertility, such that nutrient‐poor environments are dominated by slower‐growing plants, and nutrient‐rich environments are dominated by faster‐growing plants (Aerts & Chapin, 2000). This relationship is hypothesised to be a significant factor underlying global trait variation (Díaz et al., 2016; Wright et al., 2004). Life history strategy (i.e. annual or perennial) is also an important axis of growth rate variation among wild species (Garnier, 1992; Grime & Hunt, 1975), and the growth rate differences between annuals and perennials are well documented (Houghton, Thompson, & Rees, 2013). Annuals grow quickly in order to make a high investment in reproduction during their single year of life, whereas perennials grow more slowly to conserve resources for future years (Bennett, Roberts, & Wagstaff, 2012; Garnier, 1992; Iwasa, 2000). Therefore, annuals generally have a higher relative growth rate (RGR) than perennials, which is especially clear when congeneric species are compared (Garnier, 1992). Despite the dominance of slow growers in nutrient‐poor environments (Aerts & Chapin, 2000), fast‐growing species still grow faster than slow‐growing ones in infertile soil (Campbell & Grime, 1992). Fast‐growers have a high nutrient uptake capacity and a greater flexibility to alter their uptake capacity in response to nutrient availability, that is, a greater physiological plasticity (Aerts & Chapin, 2000; Garnier, Koch, Roy, & Mooney, 1989).

This study compares the responses of two barley species to a nutrient gradient, in order to investigate how nutrient availability and uptake limit vegetative growth. It aims to elucidate the relative importance of indirect limitation via carbon source strength, by measuring carbohydrate content and amino acid:sucrose ratio, and direct limitation of sink tissue expansion growth, by measuring relative growth rate (RGR) and tissue composition. The work compares an elite fast‐growing domesticated annual spring malting barley (*Hordeum vulgare* cv. NFC Tipple) with a slower‐growing wild perennial relative (*Hordeum bulbosum*). By working with species with different life history strategies, this approach uses pre‐existing variation in growth rate to probe the nature of the annual crop system. Previous work on these species during the vegetative growth stage shows significant carbon sink limitation of growth in the annual, as evidenced by a lack of plasticity of photosynthesis and allocation, and a weak growth response to elevated CO_2_ concentration (Burnett et al., 2016). In contrast, the perennial exhibits carbon source limitation, and is able to increase sink development and utilize the additional carbon available from photosynthesis under elevated CO_2_ (Burnett et al., 2016). This study investigates the role of nutrient limitation in this system, testing the alternative hypotheses that: (a) nutrients (primarily nitrogen) limit photosynthesis, thereby causing carbon limitation of growth or (b) that nutrients directly limit expansion growth. It also tests the hypothesis (c) that vegetative growth in the fast‐growing annual is more limited by its ability to take up soil nutrients (source limitation), while the slow‐growing perennial is more limited by its ability to utilize these nutrients (sink limitation).

## 2 MATERIALS AND METHODS

### 2.1 Plant material, growth conditions, and nutrient treatment

Seeds of *Hordeum vulgare* cv. NFC Tipple from the UK HGCA (2014) recommended list and *Hordeum bulbosum* (Accessions GRA1031 and GRA947) from Turkey (von Bothmer, 1996) were obtained from Syngenta and IPK Gatersleben, respectively. Data were collected until 42 days after germination, in order to focus measurements on the period of maximal vegetative growth, since previous work had found that maximum RGR occurs approximately 28 days after germination during the vegetative growth stage in these species (Burnett et al., 2016). Seeds were first germinated on wet filter paper, then transplanted to 4‐L pots filled with a 1:10 sand:vermiculite mix topped with an additional layer of sand to aid seedling root development. This mix was designed to provide a very low‐nutrient, nitrogen‐free substrate to which varying levels of nutrient solution could be added.

Plants were grown at the University of Sheffield in controlled environment plant growth chambers (BDR 16, Conviron, Isleham, UK), modified to scrub CO_2_ using soda lime to achieve the current (2015) ambient atmospheric level of 400 μmol/mol CO_2_. Plants were randomised between three chambers, with the following growth conditions: 12‐hr photoperiod with day/night temperatures of 20/18°C, 65% relative humidity, 400 μmol/mol CO_2_, and daytime light levels of 600 μmol photons m^−2^ s^−1^ to provide a daily light integral of 25.9 mol m^−2^ day^−1^.

For the first week, plants were watered daily with Reverse Osmosis water. Thereafter, plants were watered three times per week with 250 ml Long Ashton nutrient solution, applied at different concentrations (nutrient treatments): “low nutrients” (1% of Long Ashton stock solution), “medium nutrients” (20% of stock), and “high nutrients” (100% of stock). One hundred percent Long Ashton solution contains 4.00 mM potassium nitrate, 3.92 mM calcium nitrate tetrahydrate, 1.73 mM sodium dihydrogen orthophosphate, 1.49 mM magnesium sulphate heptahydrate, 0.09 mM ethylenediaminetetraacetic acid ferric monosodium salt, 1.92 μM manganese sulphate tetrahydrate, 0.35 μM zinc sulphate, 0.19 μM copper sulphate pentahydrate, 9.65 μM boric acid, 0.11 μM sodium molybdate, and 19.27 μM sodium chloride, in Reverse Osmosis water. Long Ashton's solution provides mineral nutrients in proportions appropriate to the requirements of the plant, so the treatment altered the application of all mineral nutrients in accordance with these proportions. Plants did not display visible signs of mineral deficiency or toxicity (Supporting Information Figure S1).

### 2.2 ^15^N uptake

Nitrate labelled with the ^15^N stable isotope (^15^NO_3_^−^) was fed 24 hr before the final harvest at 42 days after germination. A ^15^N‐labelled potassium nitrate and calcium nitrate (10% labelled atom) were substituted for the same mass of unlabelled potassium nitrate and calcium nitrate in the Long Ashton nutrient solution, such that the plants received their usual dose of all nutrients.

A subsample of the ground, freeze‐dried material obtained for metabolite measurements (see below) was analysed for ^15^N enrichment using an ANCA GSL 20‐20 Mass Spectrometer (Sercon PDZ Europa, Crewe, UK). Enrichment was calculated as ^15^N/(^14^N + ^15^N) and adjusted for percentage labelled atom fed, and values from control samples which did not receive ^15^N were subtracted from these data in order to give ^15^N enrichment relative to the baseline level for each organ type and nutrient treatment (baseline ^15^N of control samples did not differ between the two species).

### 2.3 Growth and allocation

Shoot area was obtained by photographing plants twice per week starting 8 days after germination, using the method described by Burnett et al. (2016). For very young plants, only one photograph was required, whereas six photographs from evenly spaced angles were used for larger plants. Shoot area was calibrated with whole‐plant dry mass using a batch of 29 additional plants, not used in the main study, which were also photographed twice weekly. These additional individuals were harvested and oven‐dried 12, 19, 26, 33, and 40 days after germination in order to calibrate shoot area to leaf area and dry mass (*r* ^2^ = 0.88), and to analyse partitioning to different organs. Biomass data for plants in the main study were predicted using this calibration. Mass‐based relative growth rate (RGR) was obtained for each individual in the main study by converting non‐destructive projected area measurements to whole‐plant dry mass using the calibration from the additional batch of destructively harvested plants and calculating the change in the natural logarithm of dry mass over time. Tillers were counted on all plants in the main study immediately prior to metabolite harvests.

Dry mass fractions of leaf, leaf sheath, and root were calculated for each oven‐dried individual from the additional batch of plants and used to calculate leaf mass ratio (LMR; kg leaf dry mass per kg plant dry mass). Mean LMR values for each species and nitrogen concentration were then used to predict LMR for plants in the main study. Specific leaf area (SLA; m^2^ leaf per kg leaf) was recorded for all plants in the main study by measuring the area and fresh mass of harvested leaves, and converting fresh mass into dry mass using the calibration obtained from oven‐dried plants. Leaf area ratio (LAR; m^2^ leaf per kg plant) for each individual in the main study was obtained by multiplying SLA by predicted LMR.

Data from the additional subset of 29 destructively harvested and oven‐dried plants were used only for LMR (Table 1) and for calibrating RGR and LMR for plants in the main study. All other data were obtained from the plants in the main study.

**Table 1:** Root:shoot, leaf mass, sheath mass, and root mass ratios in annual and perennial barley at different nutrient treatment levels

	Species	Low nutrients	Medium nutrients	High nutrients
Root:shoot ratio	Annual	1.60 ± 0.2	0.80 ± 0.2	0.54 ± 0.1
	Perennial	1.54 ± 0.3	0.83 ± 0.2	0.59 ± 0.07
Leaf mass ratio	Annual	0.33 ± 0.03	0.42 ± 0.03	0.46 ± 0.01
	Perennial	0.27 ± 0.01	0.35 ± 0.01	0.44 ± 0.04
Sheath mass ratio	Annual	0.07 ± 0.004	0.17 ± 0.03	0.24 ± 0.02
	Perennial	0.14 ± 0.05	0.20 ± 0.07	0.19 ± 0.02
Root mass ratio	Annual	0.60 ± 0.03	0.41 ± 0.06	0.30 ± 0.01
	Perennial	0.59 ± 0.05	0.45 ± 0.05	0.37 ± 0.03

Allocation to roots increases at lower nutrient supplies (*p* < 0.001) and allocation to leaves and sheaths decreases at lower nutrient supplies (*p* < 0.001). These data are obtained from the subset of 29 additional plants harvested for biomass calibration described in the Materials and Methods section. For root:shoot ratio, root mass is divided by the sum of leaf and sheath mass for each individual and the results averaged. For leaf, sheath, and root mass ratios, the ratio is the dry mass of that organ divided by the dry mass of the whole plant. 18 annuals and 11 perennials were harvested at five timepoints. Data show mean ± *SE* (at low, medium, high nutrients, annual *n* = 6, 6, 5; perennial *n* = 4, 2, 5). For brevity, age effects have not been included in this summary, but they are discussed elsewhere in the manuscript.

### 2.4 Metabolites

Metabolite harvests were carried out on plants from the main study. Harvests were carried out at 14, 28, and 42 days after germination; all these timepoints were within the vegetative growth stage. Plants were always watered 24 hr before harvest so that all samples would be equivalent with respect to watering schedule. For each nutrient level and species, one replicate from each of the three growth chambers was taken, giving a total of 18 plants at each timepoint. Due to the small size of plants harvested 14 days after germination, it was necessary to pool two individuals of the same species and nutrient treatment for each replicate, to obtain enough material for analysis. Plants were harvested within 1 hr before dawn, in order to capture pre‐dawn metabolite levels, when the diurnal concentrations of carbohydrate are expected to be minimal, following previous work (Burnett et al., 2016). Plants were separated into leaf, leaf sheath, and root, flash‐frozen in liquid nitrogen and stored at −80°C; samples were subsequently freeze‐dried prior to analysis. For small plants, the entire plant was harvested; for larger plants, representative samples of each organ from both young and old tissue were harvested. Metabolite assays were carried out as described previously (Burnett et al., 2016): glucose, fructose, sucrose, low and high degree of polymerisation (LDP and HDP) fructans, and starch were quantified using continuous enzymatic substrate assays, whilst nitrate, amino acids, and proteins were quantified using the Griess reaction, fluorescamine assay, and bicinchoninic acid assay, respectively. Elemental CHN was analysed using a 2400 Series II CHN analyser (PerkinElmer, Waltham, MA, USA). More samples were available for CHN analysis than for metabolite assays, due to the small size of some plants. Metabolites and CHN were expressed per gram carbohydrate‐corrected dry weight (CCDW), obtained by subtracting the total mass of non‐structural carbohydrate (the sum of: glucose, fructose, sucrose, LDP and HDP fructan, and starch) from the dry mass of each sample. Metabolite data are available in Supporting Information Table 1.

### 2.5 Statistical methods

All data were analysed in R (R Core Team, 2009) using Type II ANOVA. Natural logarithmic transformations were performed prior to analysis to satisfy the normality assumptions of ANOVA.

## 3 RESULTS

**Figure 1:**
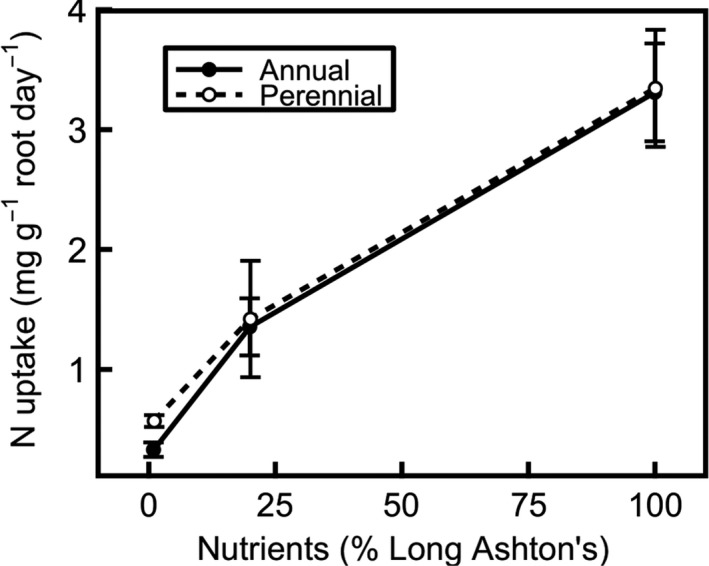
Net nitrogen uptake rate of annual (filled circles, solid line) and perennial barley (hollow circles, dashed line) decreases as nutrient treatment level is lowered (*p* < 0.001). Uptake was measured over 24 hr before harvesting plants 42 days after germination. Data show mean ± *SE* (*n* = 3)

**Figure 2:**
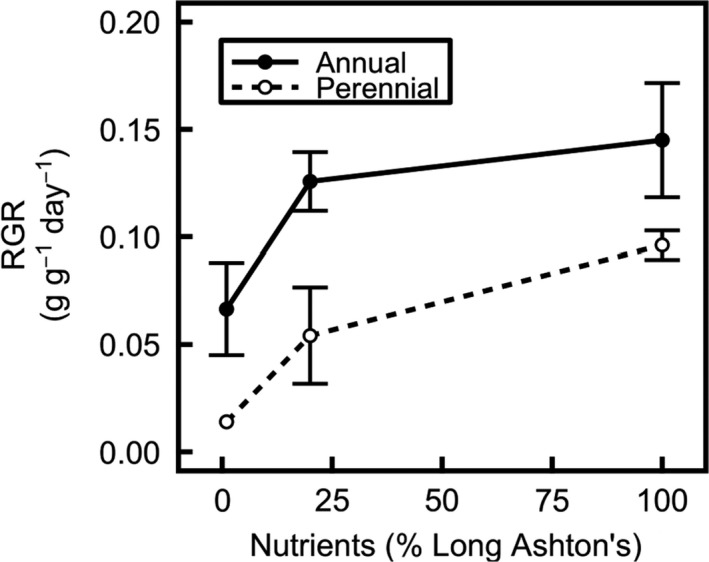
Relative growth rate (RGR, g g^−1^ day^−1^) is higher in annual (solid line) than in perennial (dashed line) barley (*p* < 0.01). Data are from plants harvested 28 and 42 days after germination. Data show mean ± *SE* (for annuals, *n* = 5, 6, 4, and for perennials, *n* = 1, 3, 3 at low, medium, and high nutrients, respectively)

Net nitrogen uptake (Figure 1) decreases significantly as nutrient treatment is lowered (*F*_2,10_ = 54.6, *p* < 0.001), but there is no significant species effect (either main effect or interaction, *p* > 0.5 in each case) on net nitrogen uptake (Figure 1). Relative growth rate (RGR; Figure 2) is consistently higher in annual than in perennial barley, suggesting that the annual invests more resources into growth than the perennial, even at low nutrient levels (significant effect of species: *F*_1,11_ = 9.77, *p* < 0.01). This shows that the nutrient use efficiency of growth is higher in the annual. RGR decreases significantly in annual and perennial barley when nutrient treatment is lowered (*F*_2,11_ = 7.98, *p* < 0.01), and this effect is especially strong when nutrients are decreased from the medium to low treatment level.

**Figure 3:**
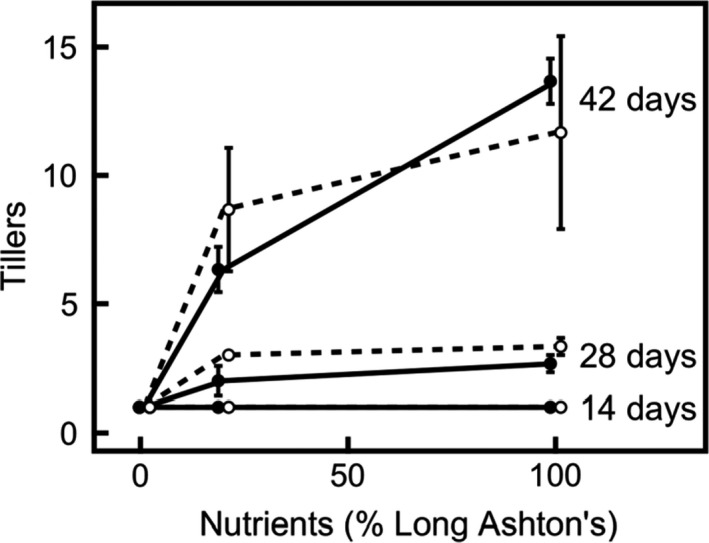
Tillering increases with nutrient treatment and this effect increases with age in both annual (filled circles, solid line) and perennial (hollow circles, dashed line) barley (*p* < 0.001). Data show mean ± *SE* (*n* = 3), for plants harvested 14, 28, and 42 days after germination; at all three ages, plants at low nutrient treatment have only one tiller. Points are offset with respect to *x*‐axis position, to increase readability

Biomass partitioning is also affected by nutrient treatment. In accordance with the change in RGR, tillering decreases substantially at low nutrient levels (Figure 3); this effect is greater in older plants (significant nutrient × age interaction for tillering: *F*_4,24_ = 13.1, *p* < 0.001). However, whilst the nutrient effect is strong, there is neither a difference between species nor a nutrient × species interaction (*p* > 0.6 in each case). Allocation to roots (Table 1) increases at lower nutrient supplies (for root:shoot ratio, *F*_1,19_ = 40.6, *p* < 0.001; for root mass ratio (RMR), *F*_1,19_ = 38.9, *p* < 0.001), but does not differ between species. In both species, sheath mass ratio and leaf mass ratio (SMR and LMR; Table 1) decrease at low nutrient levels (for sheaths, *F*_1,19_ = 25.9, *p* < 0.001; for leaves, *F*_1,19_ = 30.1, *p* < 0.001). The annual allocates more biomass to roots earlier in development and more to sheaths later in development whilst the opposite trend is seen in the perennial (significant species × age interactions: for SMR, *F*_1,19_ = 16.8, *p* < 0.001; for root:shoot ratio, *F*_1,19_ = 10.6, *p* < 0.01).

Leaf area ratio (LAR) does not differ between species, although specific leaf area (SLA) is slightly higher in the perennial (Table 2; SLA: *F*_1,45_ = 5.97, *p* < 0.05). LAR and SLA decrease with age in both species (LAR: *F*_1,45_ = 34.6, *p* < 0.001; SLA: *F*_1,45_ = 42.2, *p* < 0.001), and LAR decreases at lower nutrient treatment levels (*F*_1,45_ = 56.6, *p* < 0.001), in addition to the decrease in LMR documented above (Tables 1 and 2). Thus at lower nutrient treatment levels, proportionately less biomass is allocated to leaves, and there is a lower leaf area per unit of plant biomass.

**Table 2:** Leaf area ratio (LAR) and specific leaf area (SLA) in annual and perennial barley grown at different nutrient levels and harvested 14, 28, and 42 days after germination

	Species	Low nutrients	Medium nutrients	High nutrients
LAR at 14 days	Annual	11.3 ± 0.5	14.7 ± 1	20.0 ± 2
	Perennial	9.5 ± 0.8	16.1 ± 0.7	19.9 ± 2
LAR at 28 days	Annual	12.0 ± 0.2	13.7 ± 0.5	17.3 ± 1
	Perennial	12.3 ± 3	13.6 ± 1	16.1 ± 2
LAR at 42 days	Annual	9.8 ± 0.1	10.3 ± 0.6	12.3 ± 0.2
	Perennial	7.7 ± 0.4	10.6 ± 1	13.2 ± 0.3
SLA at 14 days	Annual	34.3 ± 1	35.0 ± 3	43.4 ± 4
	Perennial	35.1 ± 3	46.1 ± 2	45.3 ± 4
SLA at 28 days	Annual	36.5 ± 0.7	32.6 ± 1	37.6 ± 2
	Perennial	45.7 ± 12	38.9 ± 3	36.6 ± 4
SLA at 42 days	Annual	29.6 ± 0.3	24.6 ± 1	26.8 ± 0.4
	Perennial	28.6 ± 2	30.3 ± 3	30.1 ± 0.7

LAR decreases as nutrient treatment level is lowered (*p* < 0.001). SLA is higher in perennial than annual barley overall (*p* < 0.05). Both LAR and SLA are lower in older plants (*p* < 0.001). These data are for plants in the main study. LAR is expressed here as m^2^ leaf/kg plant and SLA is expressed here as m^2^ leaf/kg leaf. LAR is the product of SLA (measured on all plants in the main study at the time of metabolite harvest) and LMR (from the subset of plants harvested for biomass calibration; see Table 1). Data show mean ± *SE* (*n* = 3).

Annual plants have a greater absolute size than perennials (Table 3). Predicted biomass increases with age, especially in annuals (species × age interaction: *F*_1,415_ = 25.1, *p* < 0.001) and especially at higher nutrient levels (nutrient × age interaction: *F*_2,415_ = 48.3, *p* < 0.001). For brevity, only predicted biomass data collected before each destructive harvest are presented in Table 3, but the effects reported here are for all predicted biomass data (obtained twice weekly).

**Table 3:** Predicted biomass in annual and perennial barley grown at different nutrient levels, at 12, 22, and 40 days after germination

	Species	Low nutrients	Medium nutrients	High nutrients
12 days	Annual	0.034 ± 0.004	0.042 ± 0.01	0.052 ± 0.01
	Perennial	0.017 ± 0.003	0.062 ± 0.03	0.022 ± 0.003
22 days	Annual	0.042 ± 0.01	0.172 ± 0.02	0.228 ± 0.03
	Perennial	0.023 ± 0.01	0.056 ± 0.01	0.070 ± 0.01
40 days	Annual	0.149 ± 0.01	0.630 ± 0.09	1.367 ± 0.07
	Perennial	0.032 ± 0.01	0.236 ± 0.09	0.492 ± 0.2

Predicted biomass increases with age especially in annual barley, and especially at higher nutrient treatment levels (*p* < 0.001 for each of these interactions). These data are for plants in the main study. Biomass (g) is predicted from leaf area data, using the correlation obtained for the subset of additional plants harvested for biomass calibration. Predicted biomass is derived from photographs preceding destructive harvests (carried out at 14, 28, and 42 days after germination). Data show mean ± *SE*, with n decreasing over time due to destructive harvests (at 12 days, at low, medium, high nutrients, annual *n* = 17, 18, 18; perennial *n* = 7, 13, 15; at 22 days, at low, medium, high nutrients, annual *n* = 10, 10, 10; perennial *n* = 6, 6, 9; at 40 days, at low, medium, high nutrients, annual *n* = 5, 5, 5; perennial *n* = 3, 3, 4).

**Figure 4:**
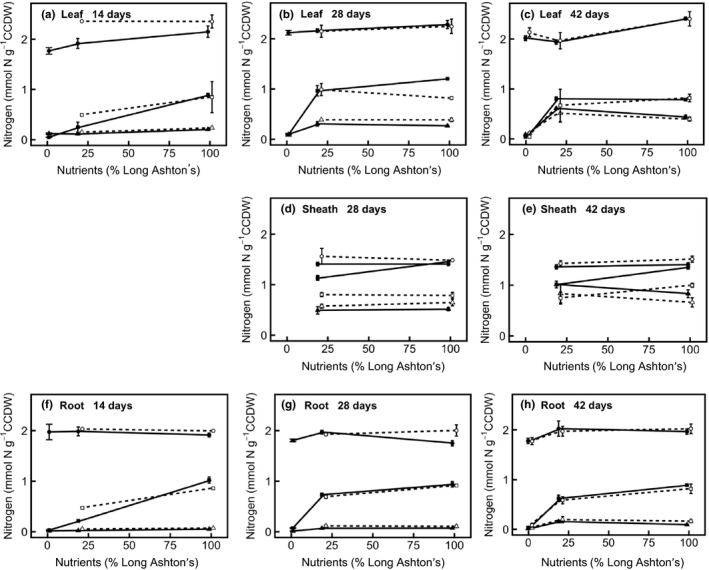
Partitioning of nitrogen resources in different organs of annual (filled symbols, solid line) and perennial (hollow symbols, dashed line) barley. Statistics are outlined in the main text. Panel shows allocation of nitrogen to protein (filled and hollow circles), free nitrate (filled and hollow squares), and free amino acids (filled and hollow triangles). All data are expressed in mmol N per gram carbohydrate‐corrected dry weight (CCDW). CCDW is obtained by subtracting the mass of total non‐structural carbohydrate (the sum of: glucose, fructose, sucrose, LDP and HDP fructan, and starch) from the dry mass of each sample. (a) Leaf 14 days after germination; (b) leaf 28 days; (c) leaf 42 days; (d) leaf sheath 28 days; (e) leaf sheath 42 days; (f) root 14 days; (g) root 28 days; (h), root 42 days. Leaf and leaf sheath tissues were pooled for the harvests at 14 days. Data show mean ± *SE* (*n* = 3). Points are offset with respect to *x*‐axis position, to increase readability

Overall, a decrease in nutrient treatment leads to a decrease in the concentration of nitrogen metabolites in annual and perennial barley (Figure 4), especially when nutrient treatment is decreased from medium to low. A comparatively smaller decrease is observed between high and medium nutrient levels, similar to the response of RGR to nutrient treatment (Figures 2 and 4). Nitrate and amino acids generally show a response to nutrient treatment level across the plant. In annual and perennial barley, with decreasing nutrient treatment, there is a significantly lower nitrate concentration in leaf (*F*_2,24_ = 64.5, *p* < 0.001), leaf sheath (*F*_1,11_ = 17.9, *p* < 0.01), and root (*F*_2,25_ = 182.7, *p* < 0.001). Nitrate in the sheath tissue is significantly higher in the annual (*F*_1,11_ = 45.9, *p* < 0.001). Amino acid concentration in leaf and root also decreases at lower nutrient treatment levels, and this effect is greater in older plants (nutrient treatment × age interaction in leaf: *F*_4,24_ = 12.1, *p* < 0.001; in root: *F*_4,25_ = 7.9, *p* < 0.001). Amino acid concentration in the sheath is not affected by nutrient treatment, but increases with age (Figure 4d,e; *F*_1,11_ = 20.8, *p* < 0.001). In the root, amino acid content is higher in perennial than annual barley (*F*_1,24_ = 26.1, *p* < 0.001). In contrast to nitrate and amino acids, protein does not show a whole‐plant response to nutrient treatment level. Protein concentration shows an overall decrease from high to low nutrient treatment levels in the leaf (Figure 4; *F*_2,25_ = 13.9, *p* < 0.001), but does not show a significant change in sheath or root.

**Figure 5:**
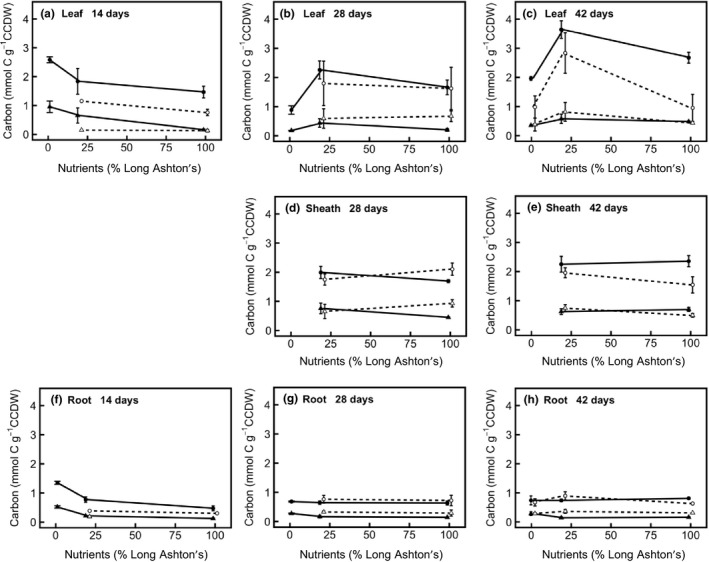
Partitioning of total non‐structural carbohydrates (TNC) in different organs of annual (filled symbols, solid line) and perennial (hollow symbols, dashed line) barley. Statistics are outlined in the main text. Panel shows TNC (filled and hollow circles) and the proportion of TNC allocated to fructans (filled and hollow triangles). TNC is the sum of glucose, fructose, sucrose, and LDP and HDP fructans. All data are expressed in mmol C per gram carbohydrate‐corrected dry weight (CCDW). CCDW is obtained by subtracting the mass of total non‐structural carbohydrate (the sum of: glucose, fructose, sucrose, LDP and HDP fructan, and starch) from the dry mass of each sample. (a) Leaf 14 days after germination; (b) leaf 28 days; (c) leaf 42 days; (d) leaf sheath 28 days; (e) leaf sheath 42 days; (f) root 14 days; (g) root 28 days; (h) root 42 days. Leaf and leaf sheath tissues were pooled for the harvests at 14 days. Data show mean ± *SE* (*n* = 3). Points are offset with respect to *x*‐axis position, to increase readability

TNC in the leaf is significantly higher in the annual (*F*_1,24_ = 14.8, *p* < 0.001), as would be expected if the annual is more carbon sink limited than the perennial. Leaf TNC is also affected by nutrient treatment (*F*_2,24_ = 6.1, *p* < 0.01): leaf TNC increases when nutrient treatment is lowered from medium to low in younger plants (Figure 5a), yet decreases at low nutrient levels in older plants (Figure 5b,c). However, contrary to expectations that carbon source limitation would increase at low nutrient levels, leaf TNC increases when nutrient treatment is lowered from high to medium, that is, leaf TNC accumulates more at medium nutrient treatment levels than at high levels. This indicates that carbon source limitation does not increase when nutrient treatment is decreased. Rather, carbon source limitation is greater at high nutrient treatment levels. Root TNC shows a significant nutrient treatment × age interaction (*F*_4,25_ = 9.3, *p* < 0.001): the increase in TNC at lower nutrient treatment is most pronounced 14 days after germination (Figure 5f).

**Figure 6:**
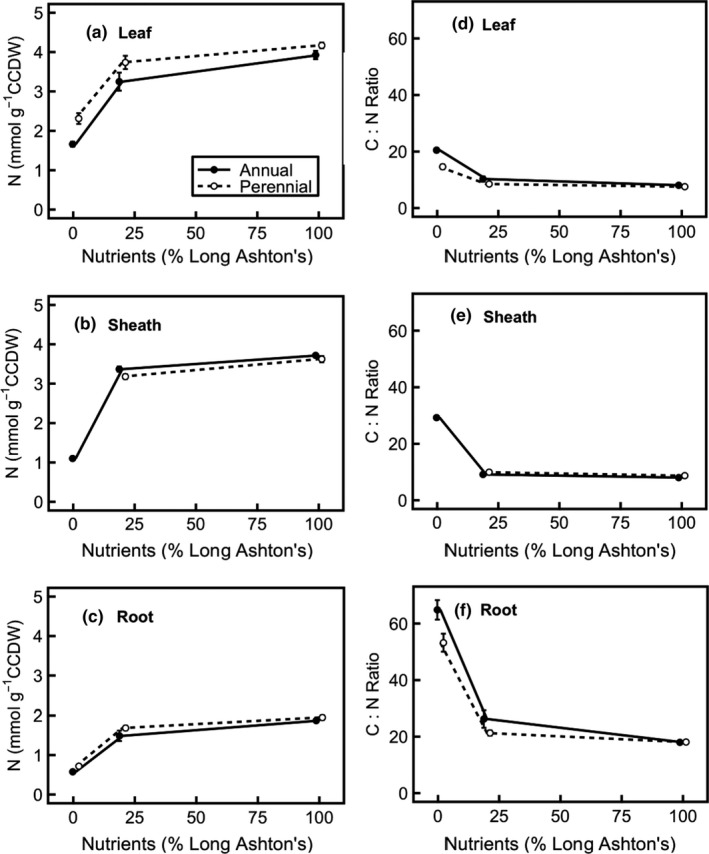
Total nitrogen concentration and carbon:nitrogen (C:N) ratio in annual (filled circles, solid line) and perennial (hollow circles, dashed line) barley. Statistics are outlined in the main text. Carbohydrate‐corrected dry weight (CCDW) was used for these elemental N and C concentrations. CCDW is obtained by subtracting the mass of total non‐structural carbohydrate (the sum of: glucose, fructose, sucrose, LDP and HDP fructan, and starch) from the dry mass of each sample. (a) Leaf nitrogen; (b) sheath nitrogen; (c) root nitrogen; (d) leaf C:N ratio; (e) sheath C:N ratio; (f) root C:N ratio. Data show mean across all ages (14, 28, 42 days after germination) due to lack of significant age effects ± *SE* (at low, medium, high nutrients, annual *n* = 9, 9, 9 in leaf and root and 1, 6, 6 for sheath; perennial *n* = 4, 7, 9 for leaf, 0, 5, 5 for sheath, and 5, 7, 9 for root). There are more samples for CHN analysis than for metabolite analysis due to the small size of some samples. Points are offset with respect to *x*‐axis position, to increase readability

Elemental N content (Figure 6a–c) decreases at lower nutrient treatment level in leaf (nutrient treatment × age interaction: *F*_4,24_ = 6.5, *p* < 0.01), sheath (nutrient treatment effect: *F*_1,11_ = 50.8, *p* < 0.001), and root (nutrient treatment × age interaction: *F*_4,25_ = 9.0, *p* < 0.001). In the leaf, elemental N content increases with age especially at medium nutrient levels; in the root, elemental N increases with age especially at low nutrient levels. Elemental N is also higher in the perennial in leaf tissue (*F*_1,24_ = 10.8, *p* < 0.01), but there is no species × nutrient treatment interaction. Carbon:nitrogen (C:N) ratio (Figure 6d–f) increases with lowering nutrient treatment in leaf (nutrient treatment × age interaction: *F*_4,24_ = 7.2, *p* < 0.001), sheath (nutrient treatment effect: *F*_1,11_ = 34.5, *p* < 0.001), and root (nutrient treatment × age interaction: *F*_4,25_ = 9.2, *p* < 0.001). In both leaf and root, C:N ratio decreases as plant age increases and this effect is strongest at low and medium nutrient treatment levels. Furthermore, C:N ratio in the leaf is higher in annual barley (*F*_1,24_ = 10.7, *p* < 0.01), and C:N ratio in the sheath is higher in perennial barley (*F*_1,11_ = 14.0, *p* < 0.01).

**Figure 7:**
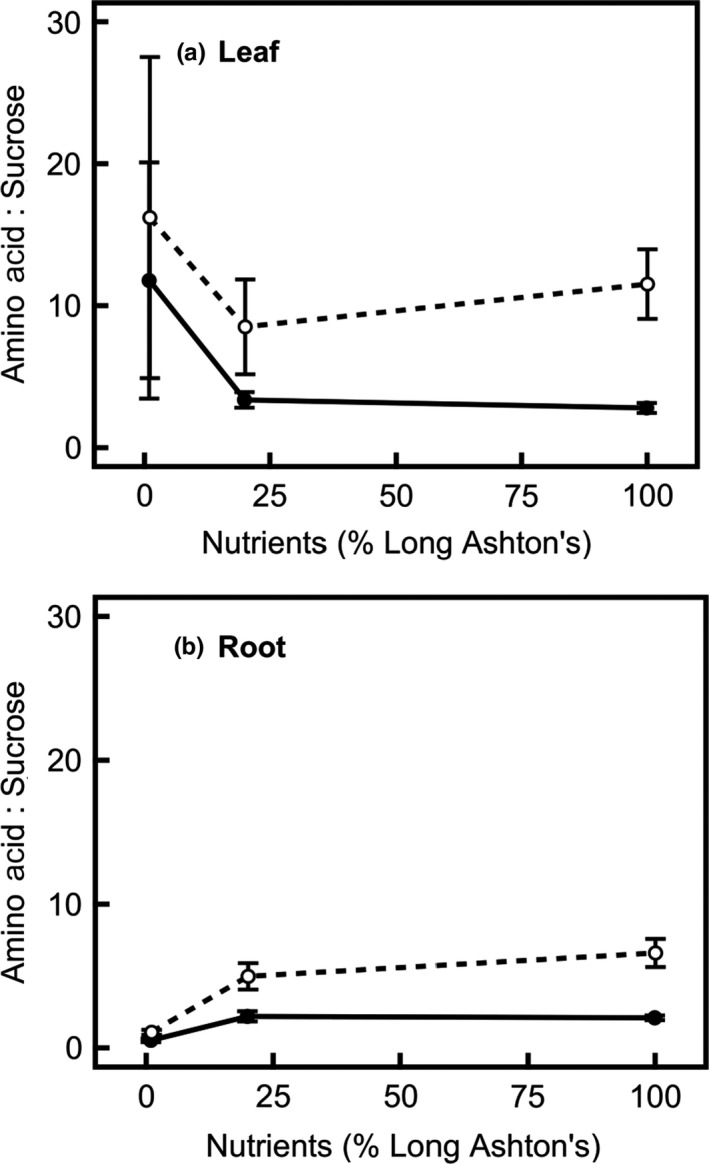
Amino acid:sucrose ratio is higher in perennial (hollow circles, dashed line) than in annual (filled circles, solid line) barley in both (a) leaf and (b) root (*p* < 0.001). In the root, amino acid:sucrose ratio increases with increasing nutrient treatment level (*p* < 0.001). Data show mean across all ages (14, 28, 42 days after germination) ± *SE* (in leaf at low, medium, high nutrients, annual *n* = 6, 8, 9, perennial *n* = 9, 8, 9; in root at low, medium, high nutrients, annual *n* = 2, 7, 8, perennial *n* = 3, 6, 7). Insufficient data were available for sheath

A high amino acid:sucrose ratio indicates that plants are carbon source limited. Perennial barley has a higher amino acid:sucrose ratio than the annual in leaf (*F*_1,22_ = 30.8, *p* < 0.001) and root (*F*_1,25_ = 43.6, *p* < 0.001; Figure 7), indicating greater carbon source limitation in perennial than annual barley. There was no significant effect of age on this ratio. Contrary to hypothesis (1), root amino acid:sucrose ratio increases at higher nutrient treatment levels (*F*_2,25_ = 64.9, *p* < 0.001), especially between low and medium nutrient levels (Figure 7b), suggesting that carbon source limitation is increased rather than decreased at high nutrient levels, rather than carbon source limitation increasing at low nutrient levels.

## 4 DISCUSSION

In this study, annual and perennial barley were grown along a nutrient gradient to examine the processes through which mineral nutrients limit growth (hypotheses 1, 2) and elucidate the relative contributions of nutrient source and sink strengths to growth in each species (3). The work focused on measurements of nitrogen uptake and concentration, since nitrogen is the primary mineral nutrient. There are multiple lines of evidence consistent with the hypothesis that nutrients directly limit expansion growth (2) rather than causing an indirect carbon limitation via photosynthesis (1). If low nutrient levels were causing carbon source limitation, via a lack of photosynthetic proteins, TNC would be expected to rise with increasing nutrient level. However, this is not always the case (Figure 5), even though leaf protein shows a small increase in the higher nutrient treatment levels (Figure 4), indicating that carbon source limitation is not alleviated by nutrients. In addition, amino acid:sucrose ratio, another indicator of carbon source limitation, increases in the root at higher nutrient treatment levels, further indicating that carbon source limitation increases at high nutrient levels rather than decreasing (Figure 7b). Consistent with this hypothesis, carbon becomes an increasingly limiting resource at higher nutrient treatment levels, and the leaf and sheath mass ratios in both species increase to compensate for this effect (Table 1). Taken together, this evidence shows that the nutrient limitation on growth is mediated by a direct constraint on expansion growth (hypothesis (2)) rather than acting via carbon limitation (hypothesis (1)).

Regarding hypothesis (3), the investment of nitrogen into growth and storage shows large decreases when nutrient treatment is decreased from a medium to a low supply, but this response is smaller when nutrients are decreased from a high to a medium nutrient supply, especially in annual barley. These results indicate a significant nutrient source limitation at low nutrient levels, but a nutrient sink limitation at high nutrient levels (Figures 2, 4 and 6). The relatively small difference in nutrient investment between medium and high nutrient levels shows that plants are nutrient sink limited under medium nutrient treatment levels. Although the annual invests more resources in overall growth (Figure 2, Table 3), the perennial is better able to increase investment in growth under high nutrient levels, in relative terms, despite its smaller size (Figure 2). This result suggests that growth in the perennial is less limited by sink development than growth in the annual, so improving nutrient sink strength will be important for improving the growth and yield of annual barley.

### 4.1 Perennial barley shows a greater growth response to nutrients

Annual and perennial barley have the same net nitrogen uptake rate (Figure 1), and both show a substantial decrease in nitrogen uptake with decreasing nutrient treatment level (Figure 1), consistent with expectations. As the nutrient treatment decreases from medium to low nutrient supply, plants show large growth and storage responses, with both the amount of plant material (Figure 2) and the concentration of nitrogen (Figure 4) decreasing significantly.

Annual barley grows faster overall (Figure 2) and is larger than the perennial (Table 3) indicating higher nutrient use efficiency of growth. RGR is more strongly limited by nutrient supply in the perennial, suggesting that the lower nutrient efficiency of growth in the perennial leads to stronger nutrient limitation when nutrient supply is restricted. The perennial shows a relatively greater growth response to nutrient level than the annual (Figure 2) and has a higher leaf nitrogen concentration (Figure 6a). This suggests that, under nitrogen‐limited conditions (i.e. the low nutrient treatment), the perennial preferentially allocates nitrogen to storage rather than growth, consistent with the hypothesis that the perennial will conserve mineral nutrients (Campbell & Grime, 1992). However, contrary to the hypothesis that growth in the perennial would be more nutrient sink limited and growth in the annual more nutrient source limited, the perennial shows a greater response to increased nutrient treatment. This indicates that growth in the perennial is in fact less nutrient sink limited than growth in the annual.

Contrary to expectations, the perennial has a higher SLA (Table 2) and higher leaf nitrogen concentration (Figure 6) than the annual—traits that are generally associated with fast‐growing species in the ecological literature (Reich et al., 2003; Wright et al., 2004). This is consistent with previous work on this species (Burnett et al., 2016), which showed carbon source limitation of growth in the perennial, since it is investing in carbon acquisition by the leaves in order to match carbon and nitrogen supply. Indeed, the higher SLA and leaf nitrogen concentration observed here for the perennial indicate that it may have the potential to be a rather fast‐growing species despite its perennial life history strategy. Potential RGR has previously been correlated with nitrogen uptake capacity (Garnier et al., 1989) and here the net nitrogen uptake rates are very similar for annual and perennial barley, although the perennial never matches the RGR of the annual. RGR (Figure 2) is higher in the annual, but LAR (the product of SLA and LMR) does not differ between species (Table 2). Whilst SLA tends to be the major contributor to LAR and thus RGR in herbaceous species (Poorter & van der Werf, 1998), some studies have found that RGR correlates with LMR rather than SLA (references within Garnier, 1992), which is consistent with these data. Furthermore, since RGR is the product of net assimilation rate (NAR) and leaf area ratio (LAR; Poorter & Remkes, 1990), the higher RGR of annual compared to perennial barley seen here is likely due to higher photosynthetic assimilation, as seen in previous work with this species (Burnett et al., 2016). Two further points of contention are: whether RGR regulates resource uptake or whether resource uptake is regulated by RGR (Garnier et al., 1989; Rodgers & Barneix, 1988); and the extent to which uptake is regulated by demand (Taulemesse, Le Gouis, Gouache, Gibon, & Allard, 2015). Indeed, nitrate itself is an important regulator of nitrogen uptake (Masclaux‐Daubresse et al., 2010). In addition to elucidating the relative contribution of nutrient source and sink to growth, a deeper understanding of the molecular drivers that underpin regulation of nutrient uptake will be an important component of improving crop nutrient use efficiency.

### 4.2 Annual and perennial barley do not store excess nitrogen as protein

Nitrate is a labile store and therefore shows a particularly strong response to nutrient treatment (Figure 4); this metabolite also shows a strong response to nitrogen treatment in wheat (Devienne, Mary, & Lamaze, 1994) and *Arabidopsis* (Tschoep et al., 2009) and, like protein, constitutes a key store for nitrogen in herbaceous plants (Millard, 1988). Here, nitrogen is taken up from the soil as nitrate, which is readily accumulated in plant tissues without the energetic cost or carbon skeletons needed to reduce nitrate to amino acids. Accumulating nitrate therefore means that nitrogen storage can increase under high nutrient treatment levels, even if carbon is limiting. Such carbon limitation could arise quickly in young plants. Consistent with this idea, amino acid accumulation is greater in older plants (Figure 4). Compared to nitrate and amino acids, protein concentration remains relatively constant at all nutrient treatment levels, except in leaf tissue (Figure 4) where a small decrease occurs when nutrient treatment level decreases. Excess nitrogen is thus preferentially stored as nitrate (short term storage) or amino acids (intermediate storage), or invested in growth, rather than being used to elevate whole‐plant protein concentration. The lack of an ontogenetic effect on leaf protein during vegetative growth is also notable (Figure 4): rather than regulating leaf protein concentration as leaves get older, barley plants create more leaf tissue and maintain the same protein concentrations; this contradicts the way in which many earth system models deal with nutrient limitation and is therefore an important avenue for further investigation.

### 4.3 High nutrient levels increase carbon source limitation

Storage of nitrogen as nitrate and amino acids suggests that plants are carbon source limited as protein synthesis requires additional carbon. Both species show a decrease in N concentration and an increase in C:N ratio as the nutrient treatment level is lowered (Figure 6). This corresponds with a shift from excess nitrogen to excess carbon in the plants (Stitt & Krapp, 1999). However, this effect is observed when nutrient treatment is decreased from medium to low, but not between high and medium nutrient levels (Figure 6) despite a high nitrate availability and uptake rate, suggesting that plants are reaching their maximum capacity for nitrogen storage at high nutrient treatment levels, and are carbon source limited. Leaf TNC data (Figure 5) also show carbon source limitation at high nutrient levels.

The higher LMR and SMR in both species at higher nutrient treatment levels (Table 1) enables greater acquisition of carbon, which becomes an increasingly limiting resource at higher nutrient treatment levels; conversely, more biomass is allocated to roots in low nutrient environments (Aerts & Chapin, 2000). Not only does LMR increase with nutrient treatment in both species, but LAR also increases (Tables 1 and 2), as observed by Garnier et al. (1989), enabling greater photosynthetic carbon acquisition since there is a greater, thinner leaf area for light harvesting. This suggests an increase in carbon source limitation at high nutrient levels. Both species show an increase in tillering as nutrient treatment is increased (Figure 3), as observed in wheat by Taulemesse et al. (2015), and especially when plants are older, facilitating a rapid increase in allocation to shoots and thus enabling greater carbon acquisition.

In addition to these structural changes, and the differences in elemental carbon and nitrogen concentrations, root amino acid:sucrose ratio increases at higher nutrient treatment levels (Figure 7b). This indicates that carbon source limitation is increasing, since the available amino acids outsupply the corresponding supply of available carbon necessary to fuel growth (Isopp, Frehner, Long, & Nösberger, 2000; Paul & Driscoll, 1997; Stitt & Krapp, 1999). The increase is particularly pronounced between low and medium nutrient treatment levels. The higher amino acid:sucrose ratio in the perennial, and lower TNC concentration indicates that the perennial is more carbon source limited than the annual (Figures 5, 6, 7); this corroborates the evidence from previous work on these species (Burnett et al., 2016).

### 4.4 Annual barley is more nutrient sink limited than perennial barley at moderate nutrient supply

In addition to the differences in the nutrient responses of each species, there are broad similarities revealed by the nutrient treatment. The treatment conditions are sufficiently strong that, at low nutrient treatment, growth in both annual and perennial barley is strongly nutrient source limited, as shown by high C:N ratios (Figure 6d–f), low nutrient uptake rates (Figure 1) and low growth rates compared to medium nutrient levels (Figure 2). At high nutrient levels, growth in the annual is more nutrient sink limited than the perennial, shown by its lower relative ability to increase growth (Figure 2). Growth at the medium nutrient supply appears to be more nutrient sink limited in annual than perennial barley, since increasing the nutrient level further has a much greater effect in the perennial.

In general, the higher RGR of annual plants arises from their large investment in leaf area and photosynthetic capacity: specific leaf area (SLA, mm^2^ leaf per gram leaf), nitrogen content, and partitioning of that nitrogen to the photosynthetic machinery are higher in the leaves of annuals, enabling greater carbon acquisition due to a larger light‐harvesting area per unit leaf mass and a greater nitrogen density associated with the photosynthetic machinery, thus facilitating high rates of carbon assimilation and faster growth (Garnier & Laurent, 1994; Grime et al., 1997; Pierce, Brusa, Vagge, & Cerabolini, 2013; Poorter & van der Werf, 1998). In contrast, perennials tend to have a lower SLA and nitrogen concentration, and invest more resources in the construction of robust, long‐lived leaves. Plants with lower SLA may also invest proportionately less leaf nitrogen in the photosynthetic machinery (Hikosaka, Hanba, Hirose, & Terashima, 1998).

The nutrient sink limitation uncovered here for annual barley at medium nutrient treatment levels implies that barley crops are unable to invest excess nutrients into growth and storage during the vegetative stage. Although nitrogen is taken up and stored as nitrate, the subsequent reduction of nitrate to organic forms of nitrogen is lacking, coupled with a lack of proportional increases in expansion growth to create a nutrient sink. Both the efficiency of nutrient acquisition and the efficiency of nutrient utilisation are important for breeders (Santa‐Maria, Moriconi, & Oliferuk, 2015), such that sink development in addition to source strength is vital for realising improved crop productivity (Burnett et al., 2016; White et al., 2016). Regarding the key mineral nutrient nitrogen, nitrogen transporters have been identified as a key target for improving the nitrogen source:sink balance (Tegeder & Masclaux‐Daubresse, 2018), whilst nitrogen allocation patterns are important for nitrogen use efficiency and yield (Perchlik & Tegeder, 2018). Additional nutrient storage, in order to build up nutrient reserves for subsequent grain filling, would require larger nutrient sinks—such as increased capacity for expansion growth—to develop during the vegetative growth stage. These could allow farmers to reduce the dosage level of fertiliser application later in development and still increase crop yield (by increasing grain size and number rather than by increasing grain nitrogen concentration), which is of interest for breeders and farmers working with malting barley (Syngenta Crop Protection, 2011). The source:sink balance of the primary mineral nutrient nitrogen is important for crop improvement (Sonnewald & Fernie, 2018), and this element is of global ecological importance (Taylor & Menge, 2018).

## 5 CONCLUSIONS

Concurrent measurements of growth and metabolite concentrations during barley development indicate that the nutrient limitation of vegetative growth is mediated via a direct effect of the nutrient supply on tissue expansion rather than an indirect limitation mediated via photosynthesis. The development of sinks for nutrient utilization becomes more limiting for growth at high nutrient supply. These results indicate the importance of sink development in mediating crop responses to nutrient supply, and highlight the importance of considering sink growth in crop models. In addition to an understanding of the contribution of source and sink strengths to growth, it will be important to investigate mechanisms linking supply and demand to effectively improve the mineral nutrient source:sink balance.

## AUTHOR CONTRIBUTIONS

ACB, AR, MR, and CPO designed the research. ACB performed the research with practical assistance from AR. ACB and MR analysed the data. ACB and CPO wrote the manuscript with contributions from AR and MR.
