# Supplementary material for: Nutrient sink limitation constrains growth in two barley species with contrasting growth strategies
Source: Plant Direct. 2018 Nov 12;2(11):e00094. doi: 10.1002/pld3.94 (PMC6508780; doi:10.1002/pld3.94)

Figure S1

Photographs of plants at 1%, 20% and 100% Long Ashton's nutrient solution. Left column shows annual barley grown at (A) 1%, (B) 20% and (C) 100% nutrient solution; right column shows perennial barley grown at (D) 1%, (E) 20% and (F) 100% nutrient solution.

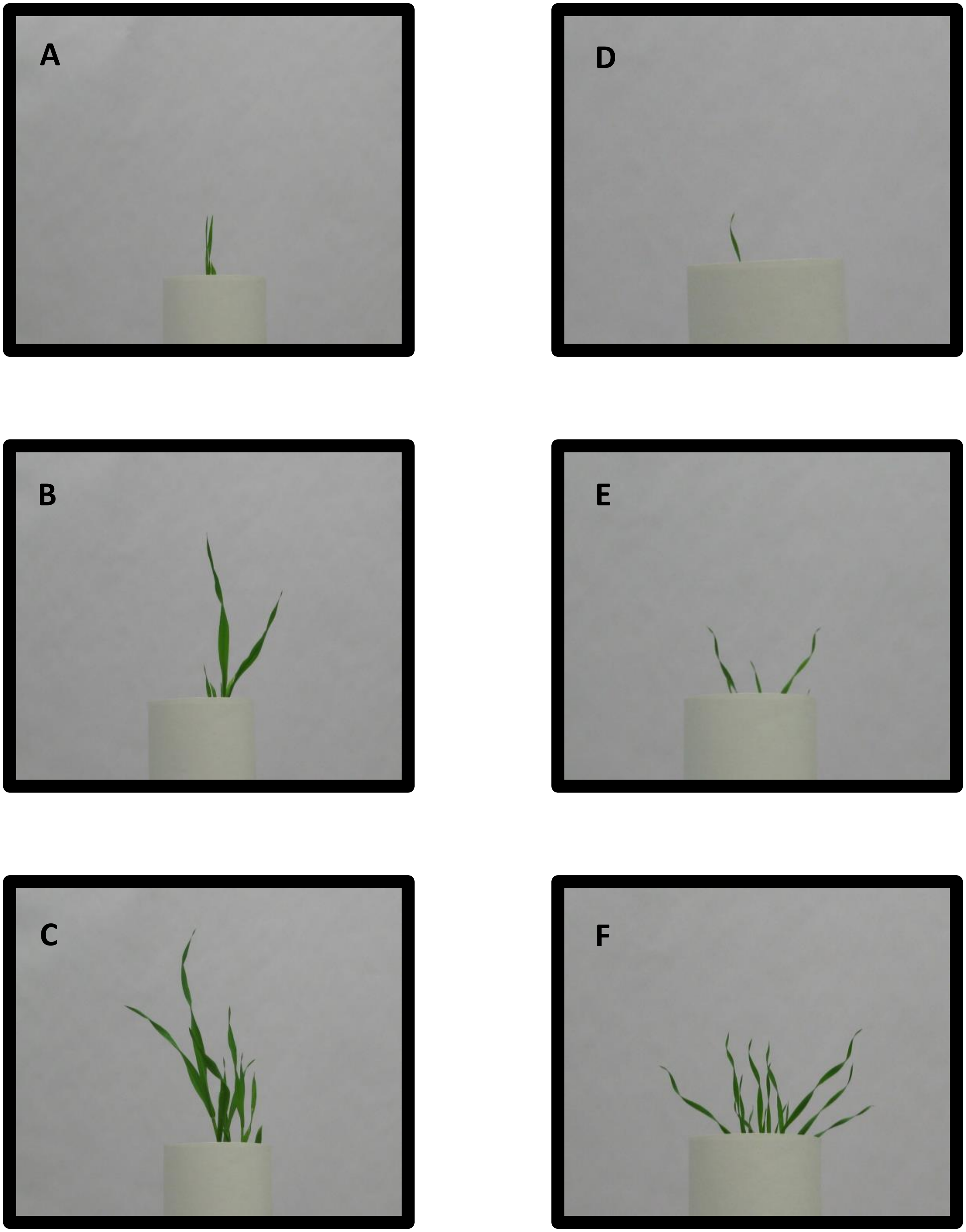

Supplement: Supplementary file 1 [file PLD3-2-e00094-s001.pdf]
