# Supplementary material for: Nutrient sink limitation constrains growth in two barley species with contrasting growth strategies
Source: Plant Direct. 2018 Nov 12;2(11):e00094. doi: 10.1002/pld3.94 (PMC6508780; doi:10.1002/pld3.94)
Supplement: Supplementary file 2 [file PLD3-2-e00094-s002.pdf]

Sample	Plant Age (days)	Growth Chamber	Plant ID	Plant 2 ID (when samples pooled)	Annual (a) / perennial (p)	Nutrient Treatment (% Long Ashton's)	Tissue
1	14	108	1	13	a	low	leaf
2	14	108	1	13	a	low	root
3	14	111	31	34	a	low	leaf
4	14	111	31	34	a	low	root
5	14	113	43	52	a	low	root
6	28	108	7		a	low	root
7	28	111	25		a	low	leaf
8	28	111	25		a	low	root
9	28	113	49		a	low	leaf
10	28	113	49		a	low	root
11	42	108	4		a	low	leaf
12	42	108	4		a	low	root
13	42	111	22		a	low	leaf
14	42	111	22		a	low	root
15	42	113	37		a	low	leaf
16	42	113	37		a	low	root
17	42	111	79		p	low	root
18	42	113	82		p	low	leaf
19	42	113	82		p	low	root
20	42	108	103		p	low	leaf
21	42	108	103		p	low	root
22	14	108	11	17	a	medium	leaf
23	14	108	11	17	a	medium	root
24	14	111	32	35	a	medium	leaf
25	14	111	32	35	a	medium	root
26	14	113	38	44	a	medium	leaf
27	14	113	80	77	p	medium	leaf
28	14	113	80	77	p	medium	root
29	28	108	14		a	medium	leaf
30	28	108	14		a	medium	root
31	28	108	14		a	medium	sheath
32	28	111	26		a	medium	leaf
33	28	111	26		a	medium	root
34	28	111	26		a	medium	sheath
35	28	113	41		a	medium	leaf
36	28	113	41		a	medium	root
37	28	113	41		a	medium	sheath
38	28	113	62		p	medium	leaf
39	28	113	62		p	medium	root

40	28	108	65		p	medium	leaf
41	28	108	65		p	medium	root
42	28	108	65		p	medium	sheath
43	28	111	83		p	medium	leaf
44	28	111	83		p	medium	root
45	28	111	83		p	medium	sheath
46	42	108	2		a	medium	leaf
47	42	108	2		a	medium	root
48	42	108	2		a	medium	sheath
49	42	111	29		a	medium	leaf
50	42	111	29		a	medium	root
51	42	111	29		a	medium	sheath
52	42	113	50		a	medium	leaf
53	42	113	50		a	medium	root
54	42	113	50		a	medium	sheath
55	42	111	86		p	medium	leaf
56	42	111	86		p	medium	root
57	42	111	86		p	medium	sheath
58	42	108	89		p	medium	leaf
59	42	108	89		p	medium	root
60	42	108	89		p	medium	sheath
61	42	113	98		p	medium	leaf
62	42	113	98		p	medium	root
63	42	113	98		p	medium	sheath
64	14	108	3	6	a	high	leaf
65	14	108	3	6	a	high	root
66	14	111	21	30	a	high	leaf
67	14	111	21	30	a	high	root
68	14	113	51	39	a	high	leaf
69	14	113	51	39	a	high	root
70	14	113	60	96	p	high	leaf
71	14	108	69	99	p	high	root
72	14	111	105	90	p	high	leaf
73	28	108	18		a	high	leaf
74	28	108	18		a	high	root
75	28	108	18		a	high	sheath
76	28	111	27		a	high	leaf
77	28	111	27		a	high	root
78	28	113	54		a	high	leaf
79	28	113	54		a	high	root
80	28	113	54		a	high	sheath
81	28	113	63		p	high	leaf
82	28	113	63		p	high	root

83	28	108	66		p	high	leaf
84	28	108	66		p	high	root
85	28	108	66		p	high	sheath
86	28	111	93		p	high	leaf
87	28	111	93		p	high	root
88	28	111	93		p	high	sheath
89	42	108	9		a	high	leaf
90	42	108	9		a	high	root
91	42	108	9		a	high	sheath
92	42	111	33		a	high	leaf
93	42	111	33		a	high	root
94	42	111	33		a	high	sheath
95	42	113	42		a	high	leaf
96	42	113	42		a	high	root
97	42	113	42		a	high	sheath
98	42	108	78		p	high	leaf
99	42	108	78		p	high	root
100	42	108	78		p	high	sheath
101	42	111	84		p	high	leaf
102	42	111	84		p	high	root
103	42	111	84		p	high	sheath
104	42	113	87		p	high	leaf
105	42	113	87		p	high	root
106	42	113	87		p	high	sheath

Sample	Glucose	Fructose	Sucrose	Short-chain Fructan	Starch	Long-chain Fructan
	nmol Glucose equivalents mg-1 carbohydrate-corrected dry weight (CCDW)					
1	75.4	73.3		44	142.4	82.3
2	33.9	48.7	65.9	50.3	3.9	38.1
3	72.3	83.6	11	94.5	92.1	98.4
4	20.8	41.3	64.7	63.3	4.1	33.4
5	46.2	75.2	14.7	45.6	0	29.1
6	5.7	18.5	40.5	26.9	4.6	23.9
7	34.5	30.5	2.7	15.6	26.8	13.8
8	3.7	9.9	48.3	20.4	4.7	24.5
9	23.7	25.6	61.8	9.1	29.8	22
10	5.2	15.3	44.8	16	4.3	25.9
11	57.5	49.1	133.2	34.8	43.8	30.4
12	7.8	20	71.9	47.8	7.1	20.2
13	36.3	27.4	146.6	24.2	57.5	34.4
14	3.9	11.9	34.6	25.8	7.2	15.1
15	37.4	33.6	154.2	22.1	34.2	31.4
16	4.6	11.6	44.7	13.3	3.6	21
17	6.2	6.6	78.9	40.9	4.1	15.1
18	21.6	15.6	5.9	9.2	31.1	17.2
19	6.2	6.1	29.7	18.6	2.9	18.5
20	23.2	19.4	34	53.4	51.3	47.4
21	12.8	16	24.4	19.3	0	31.4
22	79	61.7	21.8	71.8	57.6	59
23	15.8	40.3	45.6	16.1	3.5	22.6
24	34.4	40.4	38.2	3.6	25.1	20.9
25	11.8	30.4	32.3	7.1	6.8	25.9
26	90.4	76.9		105.7	72.4	66.6
27	25.7	22.9	56.7	5.1	63.2	19.9
28	13.9	17.9		11	1.9	19.9
29	38.3	39.6	128.4	50.1	131.6	61.3
30	11.3	30.1	41.8	21.8	0	19.2
31	79.6	65.1	0.6	143.5	71.2	43
32	18.9	20.8	216.7	24.6	73.9	55.2
33	8.1	23.3	46.3	10.6	4.6	15.7
34	78.2	73.6		65.1	54.3	39.8
35	18.6	14.2	165.2	11.1	50.9	17.1
36	7.1	21.7	41.9	3.4	4.4	13.4
37	83.5	70.5		43.9	47.1	43
38	14.7	13.8	20.2	11.3	22.3	10.1
39	5.3	9.2	16.3	4.7	8.6	40.4

40	16.6	16.5	87.9	39.5	207.5	167.2
41	19.1	19.8	45.9	28.2	4.3	25.1
42	43	51.7		92.5	81.1	56.9
43	13.9	13.9	66.7	17.8	114.9	51.1
44	16.2	21.1	49.8	43	4.4	22.5
45	67.8	61		67.8	66	0
46	27.7	26.4	353.6	57.8	133	92.6
47	10.2	17	71.1	4.9	0	26.2
48	150.1	97.1		84.5	79.6	35.9
49	61.1	59.1	347	37.1	97.5	30.9
50	9	16.5	74	8	6.7	18
51	116.4	88.2		56	69.4	65.7
52	35.5	37.8	311	38.5	61.1	32.7
53	7	11	71.8	2.8	4.3	12.2
54	109	71.6		22.8	40	48
55	44.7	35.2	183	51.8	85.1	39.8
56	11.1	7.5	48.7	29.5	3.7	17.5
57	55.2	53		108.4	71.2	41.6
58	31.4	17.3	203.7	82.3	201.6	161.1
59	14.6	15.1	76.8	59.6	6.2	23
60	123.9	50.3		65.2	68	70.6
61	14	10.9	80.8	16.1	115.9	57.4
62	18.2	19.6	43.7	25	2.8	27
63	83.2	46.8		39.3	58.6	49.3
64	46.4	38.5	81	8.7	41.8	26.2
65	9.9	14	47.4	2	10.4	14.6
66	41.4	41.8	146.4	0.8	49.3	26.4
67	7.1	18.2	32.1	6.7	4.1	17.5
68	33.7	26.4	77.7	5.4	33.5	11.1
69	4.4	10.5	37.4			
70	31.6	19.1	32.4	5.9	33.6	22.5
71	10.3	24.5	15.6			
72	25.2	24.4	11.9	4.9	33.2	9.3
73	25.2	27.8	203.7	19.6	48.1	28.7
74	13.6	33.3	42.1	10.9	7.2	15.2
75	74.7	84.2		33.9	45.5	37.1
76	20.2	15.9	85.3	11	54.1	19.9
77	5.7	18.1	34	6.6	3.3	16.8
78	14.4	12.7	181	10.4	44.4	12
79	12.9	30.2	39.1	10.5	6.3	12.6
80	78.9	98.1		17.8	37.6	59.9
81	13.5	12.1	30.4			
82	20.2	22.3	16.3	10.7	2.9	17.9

83	17.7	21.5	154.5	64.5	141.8	77.9
84	15.1	15.5	65	57.2	3.2	25.6
85	60.7	68.2	5	116.4	79.4	59
86	15.3	18.6	66.1	30	101.4	51.7
87	11.2	10.2	33.5	17.2	0.8	16.9
88	65.8	51		60	65.6	74.7
89	26.8	21.7	234.7	40	98.3	44.6
90	13.1	28.6	68.1	14.2	4.4	17.1
91	156.5	110.4		85.7	52.7	54.6
92	12.9	12.7	205.9	39	83.4	34.6
93	6.9	16.6	80.9	4.3	4.4	19.1
94	133.9	63.6	9.7	56.5	53.3	48.6
95	16.3	15.6	313	26.7	66.1	56.7
96	8.6	17.3	73.3	8	3.8	18.3
97	127.3	95.9		36.8	34.2	67.7
98	21.2	15.6	73.2	23.5	132.1	48.9
99	11.7	8.3	31.6	20.7	0	32.2
100	93.6	54.8		46.4	62.3	42.5
101	11.8	10.6	76.4			
102	15.9	15.9	19.8	18.9	5.3	35.7
103	33.7	38.5		8.8	31.7	53
104	11.9	11.2	42.3			
105	8.6	8.7	27.4	22.7	6.4	26.4
106	80.1	64.1		14.6	69.5	82.4

Sample	Protein	Amino Acids	Nitrate	Elemental C	Elemental N
	ug mg-1 CCDW	nmol mg-1 CCDW		nmol mg-1 CCDW	
1	141.1	95.8	50.8	404.9	24.5
2	175.5	13.2	42.1	441.8	7
3	131.1	75.9	48.5	400.7	21
4	141.9	9.3	15.5	433.9	8.3
5	138.4	11.2	40.4	410.4	8.5
6	141.9	8.2	65.7	446	7.1
7	166.8	69.1	117.1	396.8	21
8	140.1	11.4	63.8	454.8	7.8
9	159.7	68.1	64.1	403	27.1
10	135.4	8.7	91.1	438.5	9.7
11	148.7	51.1	31	402.6	20.7
12	143.5	15.3	44.1	441.6	10.3
13	161.7	62.8	35.1	411.6	23.4
14	129	8	34.4	417.8	6.1
15	155.5	77	62.1	394.5	26.4
16	138	9.2	30.5	465.4	7.9
17	135.9	29.7	135.6	443.8	11.9
18	171.2	81.8	54.5	388.7	31.5
19	127.5	17.5	100.4	426.6	9.1
20	157.7	83.3	31.5	399.2	30.1
21	146.4	16.2	12.3	442.4	10.2
22	141.1	62.9	184.7	382.2	31.5
23	146	14.7	201.8	444.9	12.3
24	162.6	104.6	446.2	385	37.2
25	159.6	18.9	228.2	432.4	13.3
26	138.7	71.9	116.7	397.3	30.3
27	181.5	105.1	493.5	396.5	47
28	156.5	38.6	472.3	445.7	20.4
29	166.2	263.2	990.6	388.8	52
30	152.7	52	732.3	413.3	26.1
31	104.1	313.5	1122.1	368.5	44.1
32	161.6	225.6	1130.2	379.7	52.9
33	155.9	54	790	413.7	24.7
34	107.6	444.8	1220	362.2	47.1
35	171.6	155.5	775	388.6	53
36	146.3	38	682.8	423.7	23
37	113.5	275.2	1057.3	361.1	47.7
38	176	284.6	898.6	361.7	57.8
39	149.9	60.9	659.1	419.6	25.5

40	147.2	263.4	841.1	374.9	49.9
41	144.8	78.5	662.9	442.1	22.3
42	108.1	373.5	764.4	368.1	43.7
43	173.4	266.5	1228	369.4	61.6
44	149.6	106	752.3	414.6	26.4
45	132.3	429.8	842.1	383.6	41.5
46	154.4	442.2	727.3	379	51.4
47	172.4	119.4	508.9	429.9	23.9
48	109.9	742.6	990.4	375.6	47.9
49	143	392.1	845	360.4	48.6
50	162.5	133.8	620.5	420.6	24.7
51	98.9	767.5	1039.4	373.7	50.7
52	150.8	447.3	839.6	364.1	52.5
53	133.1	76.7	737.8	432.5	24.2
54	105.6	618	1017.6	372.2	45.5
55	134	594.2	44.8	369	44.1
56	147.3	195.3	588.2	427.9	24.8
57	103.7	795	583.2	380.7	45.7
58	144.4	327.1	841.4	389.7	50
59	166.6	163.1	457.5	414.9	23.2
60	107.9	615.9	677.6	381.6	46.4
61	175.9	161.7	1125	374.4	55.8
62	141.6	53.5	706.9	423.5	22.6
63	117.8	346.4	978.1	379.9	45.3
64	148.6	145.5	912.5	360.4	48.3
65	150.3	40.5	959.7	417.6	25.6
66	169.4	134.1	826.8	375.3	49.4
67	141.3	37.1	961.2	413.8	25.8
68	178.5	149.1	935	376	52.9
69	150.2	30.4	1146	397.1	24.1
70	171.1	175.6	538.6	393	52.7
71	153.6	49.6	859.2	456.7	30.6
72	191.2	153.5	1154.9	379.7	60.1
73	164.3	191.6	1231	363	56.2
74	142.4	53.2	1049	412.8	28.2
75	105.9	385.6	1449.7	359.8	51.9
76	177.1	201.6	1218.5	379.5	61.4
77	128.5	42.5	889.8	406.3	26.9
78	186.1	168.2	1169.6	379.5	59.7
79	133.7	55	877.9	367.3	26.6
80	110.7	337.7	1473	351.3	51.9
81	180.4	260.2	863	366.5	61.6
82	170.8	41.6	953.6	391.4	26.2

83	151.6	317.1	822.4	367.5	54.8
84	141.7	96.9	884.7	442.1	26.8
85	114.3	498.2	724.2	383.6	46.6
86	187.3	234.9	776.2	395.2	56.7
87	150.2	84	899.8	400.4	27
88	114.4	407.1	847.2	371	51.2
89	184.2	344.8	829	394.7	57.8
90	143	70.1	917.8	398.9	27
91	107.1	687.5	1398	359.9	54.3
92	189.8	255.7	777.3	392.5	55.9
93	156.7	54.8	899.3	422.2	25.5
94	113.8	547.9	1391.1	374.5	52.6
95	181.9	317.3	750.8	377.1	53.1
96	154.5	71.4	851.2	395.1	26.4
97	103.8	511.5	1278.8	361.3	50.7
98	165.3	331.6	808.3	373.4	58
99	156.2	155.6	624.5	413.6	24.4
100	107.3	554.9	920.2	385.4	52.7
101	186.5	259.6	954.1	367.7	61
102	142.8	99.1	901.1	395.6	28.9
103	121.6	343.1	1050.9	367	52.7
104	203.5	241.6	698	386.3	60.2
105	168.4	94.2	921.2	404.4	26
106	120.5	495.2	1022.5	383.3	50.5
