# Supplementary material for: Nutrient sink limitation constrains growth in two barley species with contrasting growth strategies
Source: Plant Direct. 2018 Nov 12;2(11):e00094. doi: 10.1002/pld3.94 (PMC6508780; doi:10.1002/pld3.94)
Supplement: Supplementary file 3 [file PLD3-2-e00094-s003.pdf]

Response to Reviewers. Author responses are indicated with arrows (>>). Line Numbers cited by the authors refer to the tracked-changes version of the manuscript.

Reviewer #1:

I thank authors for integration of comments in the first round. I aggree that the manuscript is OK in this current version even if it does not involve any elemental analyses. However, it would be essential to understand the allocation of other elements that interact with nitrogen in aminoacid production to see a much clear figure. I appreciate the addition of last sentence to the methods section about the effects of nutrition solution. However, I still insist on seeing the plant images in a supplemental figure since science needs visaual evidence. After addition of plant pictures I believe the overall quality of the manuscript has been improved a lot compared to the original submission.

>> We have included the supplemental figure requested (Fig. S1).

Reviewer #2:

Nutrient sink limitation constrains growth in two barley species with contrasting growth strategies Angela C Burnett et. al, 2018

Specific Comments:
"Fertilizer" (American English) should be writing words for "fertilizer" which is a British English, It will be great if author change replaces this word for international readers, even though it is not considered as a mistake.

>> We leave the British/American English spellings to the discretion of the Plant Direct copyeditor.

L 106-110: With regards to the key element nitrogen, does low uptake by the root could primarily constrain growth by limiting the synthesis of photosynthetic proteins and causing carbon source limitation, or by directly limiting the synthesis of proteins and other compounds required for sink tissue expansion. Re-write the sentence with more straightforward simple sentence could be crispy for the reader with an adequate message.

>> This sentence has been restructured to improve clarity (L103-107).

L-201: Plants did not display visible signs of mineral deficiency or toxicity. A Supplementary image will be helpful for the reader to understand the necrosis or cyto-toxicity due to mineral deficiency, I would suggest if the possible author should add some images both control plants vs treated plants at different nutrients level.

>> These images have been provided in a new supplemental figure (Figure S1).

L-353: As expected if the annual is more carbon sink limited than the perennial, TNC in the leaf

is significantly higher in the annual, Re-write this sentence with direct and simple sentence will be more insightful for the reader. Also does the author indicates a single leaf only affected, as all these sections were describing singular leaf/is form rather than plural leaves/are.

>> We have restructured this sentence to improve clarity (L347-348).

L-291 to L-392: Changing leaf to leaves will be right form of description for the reader.

>> Here, we use the terms ‘leaf’, ‘leaf sheath’ and ‘root’ in the singular form to describe ‘leaf tissue’ or ‘leaf material’, etc (as originally outlined in the Methods, L263, which states that plants were separated into these three components). We believe this to be a standard way of referring to different parts of the plant and we trust that it is acceptable to the Editor.

Table 1, 2, 3: I would add two lines on the top and bottom of each table for better looks, as it stands like an orphan table format. Also, I would better remove abbreviated form such as LMR, SMR, RMR from table 1) or keep only the abbreviated form and describe them as a footnote.
Table 2: footnotes description required abbreviated form LAR, SLA.
Table 3: Biomass at the description in place of age will be good enough, the author doesn't need to write biomass at 12 days etc, rather than simply write 12 days, 22 days, and 40 days will be enough.

>> We have made all the suggested edits to the tables
